# A High Precision Terahertz Wave Image Reconstruction Algorithm

**DOI:** 10.3390/s16071139

**Published:** 2016-07-22

**Authors:** Qijia Guo, Tianying Chang, Guoshuai Geng, Chengyan Jia, Hong-Liang Cui

**Affiliations:** 1 School of Instrumentation Science and Electrical Engineering, Jilin University, Changchun 130012, China; gqj2013@gmail.com (Q.G.); genggs@foxmail.com (G.G.); chengyan_jia@163.com (C.J.); hcui@jlu.edu.cn (H.-L.C.); 2 Institute of Automation, Shandong Academy of Sciences, Jinan 250014, China

**Keywords:** Phase-shift Migration Algorithm, terahertz wave image, high precision image reconstruction

## Abstract

With the development of terahertz (THz) technology, the applications of this spectrum have become increasingly wide-ranging, in areas such as non-destructive testing, security applications and medical scanning, in which one of the most important methods is imaging. Unlike remote sensing applications, THz imaging features sources of array elements that are almost always supposed to be spherical wave radiators, including single antennae. As such, well-developed methodologies such as Range-Doppler Algorithm (RDA) are not directly applicable in such near-range situations. The Back Projection Algorithm (BPA) can provide products of high precision at the the cost of a high computational burden, while the Range Migration Algorithm (RMA) sacrifices the quality of images for efficiency. The Phase-shift Migration Algorithm (PMA) is a good alternative, the features of which combine both of the classical algorithms mentioned above. In this research, it is used for mechanical scanning, and is extended to array imaging for the first time. In addition, the performances of PMA are studied in detail in contrast to BPA and RMA. It is demonstrated in our simulations and experiments described herein that the algorithm can reconstruct images with high precision.

## 1. Introduction

THz imaging technology is widely used in nondestructive testing (NDT) [1,2,3], security screening [4,5,6,7,8,9,10], and medical scanning [11,12,13,14]. One typical THz imaging mode is based on the time-domain spectroscopic (TDS) approach, which is predicated on simultaneous determination of the field amplitude, phase and polarization [15]. It has been widely used, and its performance has been highly enhanced by researchers over the years [16,17]. In order to improve the spatial resolution to that beyond the realm of the diffraction limit, THz near-field technology with detecting probes of subwavelength aperture size should be considered [15,18,19]. However it may not be practical in some situations, such as in human screening scenarios, where, a detector array would be a workable alternative. In particular, charge-coupled devices (CCDs) in the THz range (5–13 THz) [20], and bolometers [21] with much higher responsivities in the THz frequency range are available and can be configured to suit specific imaging applications. Reading out online will be allowed in the form of single-shot recording at a pixel size of a few micrometers. Thus it will be preferred for real-time systems. However, the budget and complexity of a large-sized system are invariably determining factors in some applications, e.g., in a human THz imager with a size of 1 m × 0.5 m, such a detector array might not be practical. Another mode pertaining to conventional optics and microwaves [22] is readily deployable without drastic modifications to realize image reconstruction: an antenna array could be utilized whose elements can be reduced substantially by the employment of the multi-input/multi-output (MIMO) technology [23,24,25], or the sparse array technology [24,25,26,27]. Moreover, as the frequency band we considered in the experiments is in the millimeter wave region (W-band, 75–110 GHz) of the electromagnetic spectrum, which is in the low THz region of frequency, the method of three-dimensional electromagnetic imaging is available as well.

The problem of electromagnetic imaging is essentially an inverse problem of electromagnetic field scattering [28], governed by the scalar Helmholtz equation, subject to the proper initial and boundary conditions. However, the nonlinearity of this equation makes it difficult to obtain an analytical solution. Fortunately for most practical problems a numerical solution is sufficient. However, the process of iterations always tends to kill the potential of real-time image reconstruction. Therefore a variety of approximations were proposed in which Born approximation [29,30] is the most widely used in image reconstruction technology. Under such approximations the scalar Helmholtz equations become linear, which can be solved in conventional of ways.

In this study, image reconstruction is realized by digital signals only. Thus the objective is to transform complex signals into images, two-dimensional (2D) or three-dimensional (3D). A direct method is to form an equation through samples from each channel according to linearized Helmholtz equations, which is always an ill-posed problem, so regulation of the inverse problem is an important tool for this kind of processing [31]. For example, the simplest regulation method is Tikhonov regulation [32], which is still widely used to date for its efficiency. However, it is obvious that Tikhonov regulation sacrifices precision for robustness. This may negatively impact the quality of the resulting images. Another primary type of regulation applies a recursive process, which may be more precise but more computationally intensive. What perhaps is more troublesome is the data volume involved therein. In order to achieve a high quality image, the parameter matrix is always densely meshed into a grid, and an incredible amount of RAM is essential, especially in 3D cases. Interestingly compressive sampling technology [33,34] is famous for its low sampling rate, compared with that required by the Nyquist sampling rate, which eases the burden of sampling. This feature is quite suitable for some special occasions, such as those acquired with synthetic aperture radar by high speed aircrafts [35]. Another advantage of compressive sensing in image reconstruction is that super resolution can be realized through it, which means resolution beyond the Rayleigh criterion, and resultant better focused images [36,37].

Although methods of regulation and compressive sensing feature in some critical points, matched filtering still plays a major role in imaging applications. The primary reasons may be its robustness and relatively low computational burden. Firstly, matched filtering was deduced from the principle of maximizing signal to noise ratio, which seems to perform well against noise. Secondly, as long as the scenario conformed, no other parameters are required. Therefore it is broadly applicable. Thirdly, matched filtering is free of any searching iterations, consequently it outperforms all the other algorithms in efficiency, especially when Fast Fourier Transformation (FFT) is available.

Image reconstruction using matched filtering algorithms can generally be divided into two classes: time domain algorithms and frequency domain ones. In fact, the distinctions between them may be ascertained from how and when to perform spectral analysis, and how to make some approximations to reduce the cost of computation without impacting on the image quality too much. For example, the Range Doppler algorithm (RDA) [28] can be relegated to the frequency domain, since compensations are made in frequency domain, whereas Back Projection algorithm (BPA) [38,39] refers to a time-domain algorithm. As frequency domain algorithms take advantage of FFT, pulse compression processing can be implemented efficiently. Thus they are preferred in the cases of real-time systems. Range Migration algorithm (RMA) [28,40] relies on fewer approximations than RDA, which makes it efficient and relatively more precise than RDA. However interpolation errors cannot be avoided, and cause defocusing in range direction. BPA applies no approximations, but consumes unacceptable amount of computational resources. In practice efficiency will always be in contradiction with precision, and some kind of compromise has to be struck. However, the phase-shift migration algorithm (PMA) [41,42] can be an appropriate alternative which retains the high precision of BPA, while being free of interpolation errors. It has been applied in image reconstruction of targets under the illumination of THz Gaussian beams [43,44], as such, it will be reasonable for quasi-optical systems. However when it comes to the scenario of the multistatic case, the angular divergence of the beam, which is 120 degrees in our work, will be much larger than 30 degrees considered previously [45]. Thus the precision of Gaussian beam approximation will no longer obtain. In the present study, PMA is applied to THz imaging reconstruction under the monostatic as well as the spherical wave assumption. PMA is then extended to the multistatic case for the first time, and made available in the simulation. Finally, the performance of PMA is studied in comparison with BPA and RMA in detail in the simulations and experiments.

## 2. Methods

### 2.1. Monostatic Case

**Figure 1:**
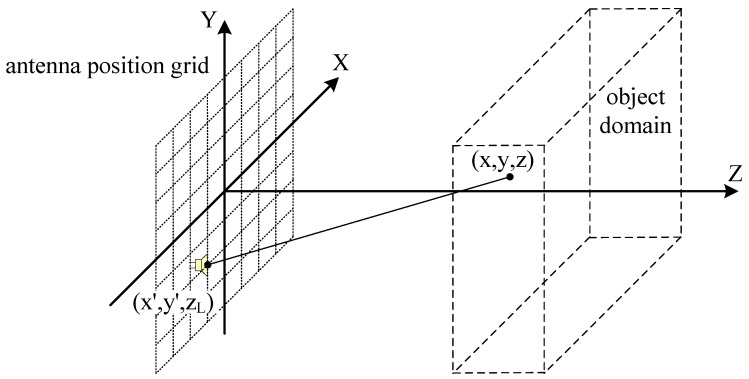
The setup of single antenna scanning scenario.

In order to reconstruct a 3D image, one could follow a number of pathways, one of which being to apply a 2D scanning aperture. The simplest scenario can be a planar scanning aperture as depicted in Figure 1. The dashed grid forms the antenna track during scanning, while each node indicates a sample position, which means equal interval sampling along *y*-axis and *z*-axis. Targets to be scanned shall be located in the dashed rectangular box area, which will be the volume to be focused.

As mentioned above, the image reconstruction process is naturally an inverse problem of electromagnetic field scattering. Therefore Helmholtz equation solved with the Born approximation is a primary part in matched filtering algorithms. In addition, it is found that if attenuation in free space is ignored, it will not impact too much in the image reconstructed. This approximation could be reasoned as follows: if the distance between the source and the observation points do not vary too much (*z* is a constant), only variations on the *x*-*y* plane in the object volume are taken into account. Additionally, the phase factors of the echoes play a major role when coherent summations are performed in our imaging scenario. Of course, the final product will just be a reference image to some extent, and each voxel presents the complex reflection coefficient of a position. Suppose the antenna is positioned at $\left(x,y,z_{L}\right)$, while a scatter point of the target is positioned at (x,y,z). Thus the objective of the imaging task is to reconstruct the reflection coefficient of (x,y,z), which is presented as p(x,y,z). Here the antenna positions form a planar array, and it is always assumed to be parallel to one of the coordinate planes, whose third coordinate component is supposed to be $z_{L}$ here. According to the assumptions made above, the system response can be expressed as [6]:

(1) $$s\left(x\prime ,y\prime ,k\right)=\underset{x,y,z}{∭}p\left(x,y,z\right)e^{-j2k\sqrt{{\left(x-x\prime \right)}^{2}+{\left(y-y\prime \right)}^{2}+{\left(z-z_{L}\right)}^{2}}}dxdydz$$

where *k* presents the wavenumber of the signal used, and X, Y and Z span the domain to be imaged, which is represented by the dashed rectangular box in Figure 1. Here the antenna pattern has not been considered. This is because antenna pattern and attenuation in free space will affect the amplitude of each voxel in approximately the same manner, whereas the phase of each voxel should be the main differentiator in this algorithm, just like in the case of synthetic aperture technology.

A 2D Fourier transformation is performed to both sides of Equation (1), after which we get:

(2) $$\begin{array}{cc} S\left(k_{x\prime },k_{y\prime },k\right) & =\int_{z}dz\iint_{x,y}\;\iint_{x\prime ,y\prime }p\left(x,y,z\right)e^{-j2k\sqrt{{\left(x-x\prime \right)}^{2}+{\left(y-y\prime \right)}^{2}+{\left(z-z_{L}\right)}^{2}}}e^{-jk_{x\prime }x\prime }e^{-jk_{y\prime }y\prime }dx\prime dy\prime dxdy\\ & =\int_{z}dz\iint_{x,y}p\left(x,y,z\right)e^{-jk_{x\prime }x}e^{-jk_{y\prime }y}e^{-jk_{z}\left(z-z_{L}\right)}dxdy\\ & =\int_{z}P\left(k_{x\prime },k_{y\prime },z\right)e^{-jk_{z}\left(z-z_{L}\right)}dz \end{array}$$

where $S\left(k_{x\prime },k_{y\prime },k\right)$ is the 2D Fourier transformation(2DFT) of $s\left(x^{\prime },y,k\right)$ relative to the position variables x and y, and $P\left(k_{x\prime },k_{y\prime },z\right)$ is the 2DFT of p(x,y,z) relative to x and y. $k_{z}$ is the wavenumber which is introduced to simplify the formula, and it is expressed as:

(3) $$k_{z}=\sqrt{4k^{2}-k_{x\prime }^{2}-k_{y\prime }^{2}}$$

According to the theory of matched filter, Equation (2) can be transformed to:

(4) $$P\left(k_{x\prime },k_{y\prime },z\right)=\int_{k}S\left(k_{x\prime },k_{y\prime },k\right)e^{jk_{z}\left(z-z_{L}\right)}dk$$

So if the signal and the echo of frequency domain is known, the reflection coefficient function p(x,y,z) will be obtained from:

(5) $$\begin{array}{cc} p\left(x,y,z\right) & =\iint_{k_{x\prime },k_{y\prime }}P\left(k_{x\prime },k_{y\prime },z\right)e^{jk_{x\prime }x}e^{jk_{y\prime }y}dk_{x\prime }dk_{y\prime }\\ & =IFT_{2D}\left[P\left(k_{x\prime },k_{y\prime },z\right)\right] \end{array}$$

Perhaps it has been noticed that $S\left(k_{x\prime },k_{y\prime },k\right)$ cannot be obtained from the echoes directly. It will be natural to conclude that S(x′,y,k) can be expressed as:

(6) $$S\left(k_{x\prime },k_{y\prime },k\right)=\frac{E\left(k_{x\prime },k_{y\prime },k\right)}{o\left(k\right)}$$

wherein E(x′,y′,k) refers to the received signal at the location $\left(x^{\prime },y^{\prime }\right)$ in wavenumber domain, and o(k) refers to the transmitted signal in wavenumber domain.

Equation (4) is a critical step to complete the pulse compression along the range direction. If discrete signals are obtained, Equation (4) will be realized in the form:

(7) $$P\left(k_{x\prime },k_{y\prime },z\right)=\sum_{k_{i}}S\left(k_{x\prime },k_{y\prime },k_{i}\right)e^{j\sqrt{4k_{i}^{2}-k_{x\prime }^{2}-k_{y\prime }^{2}}\left(z-z_{L}\right)}\Delta k$$

In Equation (7), $k_{i}$ is the sample in wavenumber domain. Δk is the sample interval of wavenumber *k*.

**Figure 2:**
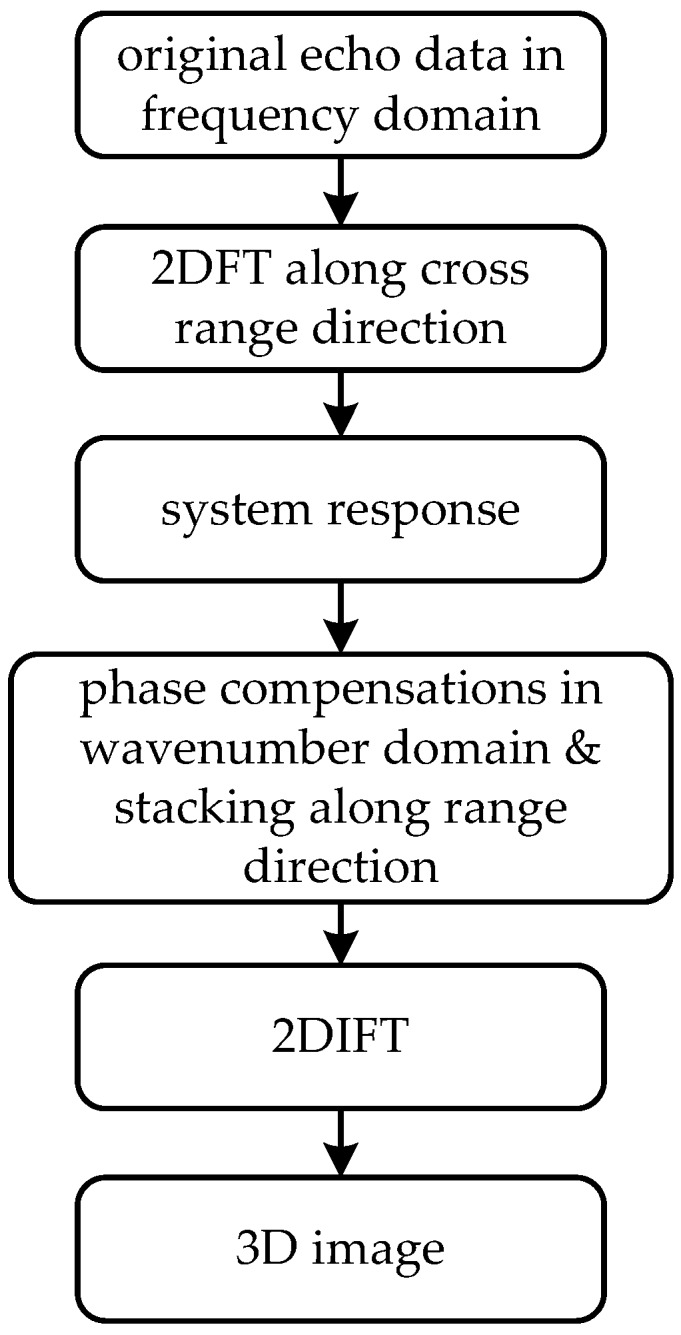
Block diagram of PMA for monostatic case.

In summary, the whole algorithm can be realized following the steps as shown in Figure 2. Firstly, the signals received should be transformed to frequency (wavenumber) domain. The echoes are often baseband complex signals. If only real signals are available, Hilbert Transformation should be performed. Then according to Equation (2), 2DFT will be performed along both cross range directions. If the excitation source is not a Dirac delta function, which is always the case, the system response must be calculated from Equation (6). The pulse compression along the range direction is realized by Equation (7). In fact, Δk is an irrelevant factor in the process that can be ignored. Finally, 2DFT and 2DIFT will be realized by FFT.

### 2.2. Multistatic Case

**Figure 3:**
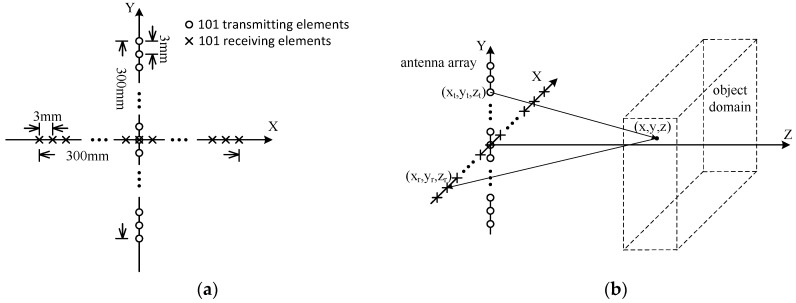
The array geometry and the setup of the experiment for multiple input and output elements: (**a**) the plus array geometry; (**b**) the setup of the experiment.

In the foregoing consideration, only one antenna is applied to scan targets mechanically. It will take much longer time than electronic scanning, which can be realized by Multiple Input Multiple Output (MIMO) or Phased Array (PA) technology. When MIMO technology is used, the corresponding scenario called the multistatic case here, the amount of antenna elements are greatly reduced than the monostatic case. The array geometry, which is called a plus array [46], and the setup of the experiment, are demonstrated schematically in Figure 3.

Suppose the locations of an arbitrary transmitting element and its corresponding receiving element are $\left(x_{t},y_{t},z_{t}\right)$, and $\left(x_{r},y_{r},z_{r}\right)$, respectively, as shown in Figure 3b, similar to Equation (1), the system response in the multistatic case of a scattering problem can be described by:

(8) $$\begin{array}{cc} s\left(x_{t},y_{t},x_{r},y_{r},k\right) & =\underset{x,y,z}{∭}p\left(x,y,z\right)e^{-jk\sqrt{{\left(x-x_{t}\right)}^{2}+{\left(y-y_{t}\right)}^{2}+{\left(z-z_{t}\right)}^{2}}}\\ & \times e^{-jk\sqrt{{\left(x-x_{r}\right)}^{2}+{\left(y-y_{r}\right)}^{2}+{\left(z-z_{r}\right)}^{2}}}dxdydz \end{array}$$

In Equation (8), the reflection coefficient function p(x,y,z) is the objective to be imaged. Compared to Equation (1), it is found that the system response is a 5D matrix. Different groups of transmitting and receiving antennas increase the dimensionality of data, which can be interpreted as the increase of degrees of freedom. That of course is one of the salient features of MIMO technology.

Then 4DFT will be performed on both sides of Equation (8) along the four cross range directions, thereafter we obtain:

(9) $$S\left(k_{xt},k_{yt},k_{xr},k_{yr},k\right)=\underset{x,y,z}{∭}p\left(x,y,z\right)F_{4D}\left[g_{t}\left(x_{t},y_{t}\right)\cdot g_{r}\left(x_{r},x_{r}\right)\right]dxdydz$$

where the operator $FT_{4D}\left[\ldots \right]$ means 4DFT of the first four dimensions of the data, with the functions:

(10) $$g_{t}\left(x_{t},y_{t}\right)=e^{-jk\sqrt{{\left(x-x_{t}\right)}^{2}+{\left(y-y_{t}\right)}^{2}+{\left(z-z_{t}\right)}^{2}}}$$

(11) $$g_{r}\left(x_{r},x_{r}\right)=e^{-jk\sqrt{{\left(x-x_{r}\right)}^{2}+{\left(y-y_{r}\right)}^{2}+{\left(z-z_{r}\right)}^{2}}}$$

As $g_{t}\left(x_{t},y_{t}\right)$ and $g_{r}\left(x_{r},y_{r}\right)$ have no coupling between their integral variables, it follows that:

(12) $$\begin{array}{cc} F_{4D}\left[g_{t}\left(x_{t},y_{t}\right)\cdot g_{r}\left(x_{r},x_{r}\right)\right] & =F_{2D}\left[g_{t}\left(x_{t},y_{t}\right)\right]\cdot F_{2D}[g_{r}\left(x_{r},x_{r}\right)]\\ & =G_{t}\left(k_{xt},k_{yt},k\right)\cdot G_{r}\left(k_{xr},k_{yr},k\right) \end{array}$$

The functions $G_{t}\left(k_{xt},k_{yt},k\right)$ and $G_{r}\left(k_{xr},k_{yr},k\right)$ can be expressed as:

(13) $$G_{t}\left(k_{xt},k_{yt},k\right){=e}^{-j\sqrt{k^{2}-k_{xt}^{2}-k_{yt}^{2}}\left(z-z_{t}\right)-jk_{xt}x-jk_{yt}y}$$

(14) $$G_{r}\left(k_{xr},k_{yr},k\right){=e}^{-j\sqrt{k^{2}-k_{xr}^{2}-k_{yr}^{2}}\left(z-z_{r}\right)-jk_{xr}x-jk_{yr}y}$$

Substitute Equations (12)–(14) into Equation (9), it is recast in the form:

(15) $$\begin{array}{cc} S\left(k_{xt},k_{yt},k_{xr},k_{yr},k\right) & =e^{j\sqrt{k^{2}-k_{xt}^{2}-k_{yt}^{2}}z_{t}+j\sqrt{k^{2}-k_{xr}^{2}-k_{yr}^{2}}z_{r}}\\ & \times \underset{x,y,z}{∭}p\left(x,y,z\right)e^{-jk_{x}x}e^{-jk_{y}y}e^{-jk_{z}z}dxdydz \end{array}$$

For convenience, a new set of variables $k_{x}$, $k_{y}$ and $k_{z}$ are introduced, defined as:

(16) $$k_{x}=k_{xt}+k_{xr}$$

(17) $$k_{y}=k_{yt}+k_{yr}$$

(18) $$k_{z}=\sqrt{k^{2}-k_{xt}^{2}-k_{yt}^{2}}+\sqrt{k^{2}-k_{xr}^{2}-k_{yr}^{2}}$$

If $P\left(k_{x},k_{y},z\right)$ expresses the 2DFT of p(x,y,z), it will be given as:

(19) $$P\left(k_{x},k_{y},z\right)=\iint_{x,y}p\left(x,y,z\right)e^{-jk_{x}x}e^{-jk_{y}y}dxdy$$

Substituting Equation (19) into Equation (15), and we will get:

(20) $$\begin{array}{cc} S\left(k_{x},k_{y},k\right) & =e^{j\sqrt{k^{2}-k_{xt}^{2}-k_{yt}^{2}}z_{t}+j\sqrt{k^{2}-k_{xr}^{2}-k_{yr}^{2}}z_{r}}\\ & \times \int_{z}P\left(k_{x},k_{y},z\right)e^{-jk_{z}z}dz \end{array}$$

According to the theory of matched filter, the formulas:

(21) $$\begin{array}{cc} P\left(k_{x},k_{y},z\right) & =e^{-j\sqrt{k^{2}-k_{xt}^{2}-k_{yt}^{2}}z_{t}-j\sqrt{k^{2}-k_{xr}^{2}-k_{yr}^{2}}z_{r}}\\ & \times \int_{k}S\left(k_{x},k_{y},k\right)e^{jk_{z}z}dk \end{array}$$

and:

(22) $$p\left(x,y,z\right)=\iint_{k_{x},k_{y}}P\left(k_{x},k_{y},z\right)e^{jk_{x}x}e^{jk_{y}y}dk_{x}dk_{y}$$

together reconstruct the final 3D image.

**Figure 4:**
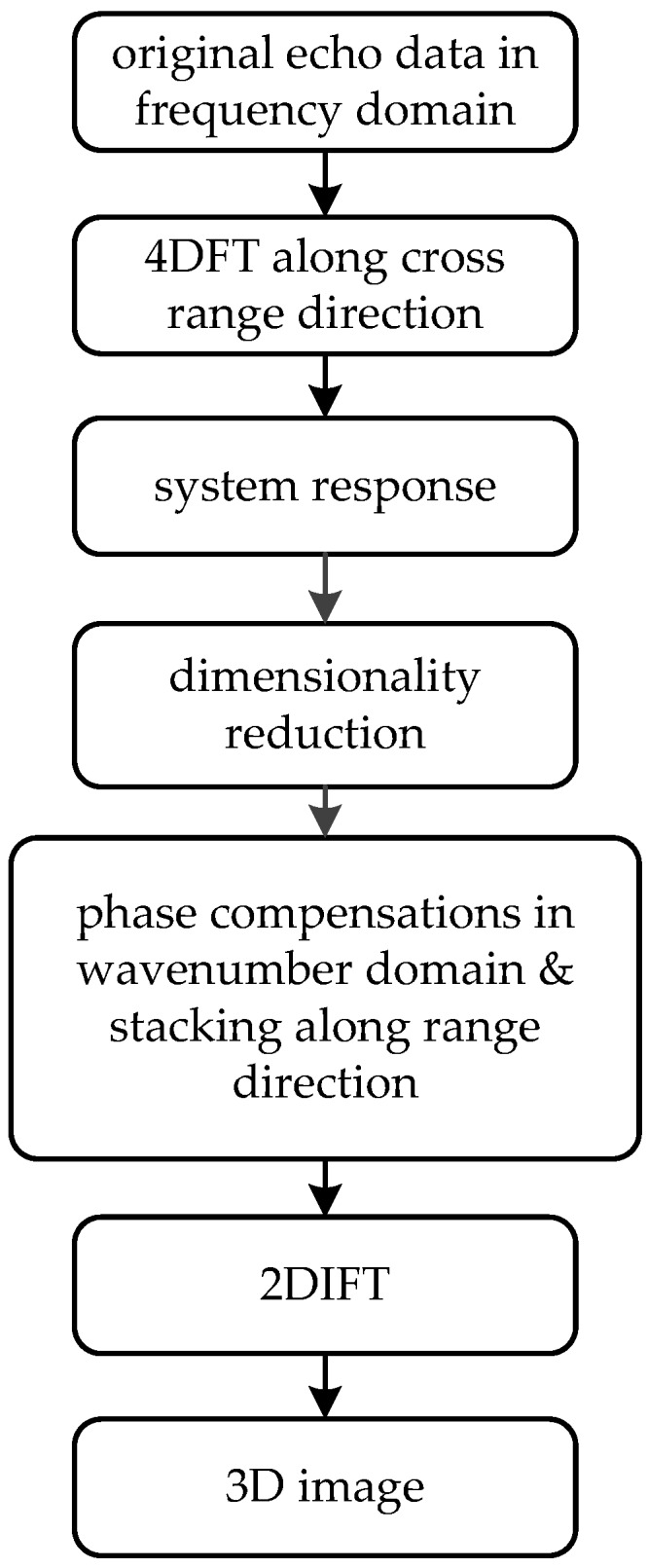
Block diagram of PMA for array imaging.

The corresponding block diagram of the process can be summarized as shown in Figure 4. Compared to Figure 2, two main issues should be noticed:

1. Phase compensations will be taken according to Equation (21), and when the array is a planar one, which is the case in Figure 3b it is always assumed $z_{t}=0$ and $z_{r}=0$ for convenience;
2. Dimensionality reduction means $S\left(k_{xt},k_{yt},k_{xr},k_{yr},k\right)$ will be transformed into $S\left(k_{x},k_{y},k\right)$, where a 5D data matrix is reduced to a 3D one. This is performed according to Equations (16) and (17).

### 2.3. Discussion on Sampling Criteria and Spatial Resolution

According to the Nyquist sampling theorem, the sensors must sample “close” enough to cover the support region of the wavenumber components. Otherwise, it will suffer from spectrum aliasing in frequency domain.

The derivation of the sampling criteria is presented in Appendix A. According to Equations (A5)–(A8), the spatial sampling interval must satisfy:

(23) Δus≤λmin(Lus+Du)24+R02Lus+Du

where u can be replaced by *x* or y and s can be replaced by t or r. In Equation (9), *L_us_* refers to the array’s aperture size of the transmitting/receiving elements in the u-direction, and *R*_0_ is the closest distance between the antenna array and the target’s front surface. Of course, if equivalent elements are considered, the sampling requirement in the same direction may become less stringent.

The spatial resolution for the multistatic case is discussed in Appendix A, and can be summarized as follows:

(24) $$\rho_{u}=\frac{\lambda_{c}R_{0}}{L_{ut}+L_{ur}}$$

where u represents the cross range direction, which can be replaced by x or y. $\lambda_{c}$ is the center wavelength. Here the sampling criteria and resolution are discussed only for the multistatic case. As the monostatic case can be deduced easily from the equations above, or be found in other papers [6,47], it will not be discussed here.

## 3. Simulation Results

In this section, two sample simulations will be undertaken to prove the effectiveness of our algorithm. To describe its performance objectively, two classical algorithms, RMA and BPA, will be introduced to provide contrasts. In the following, the coordinate system adopted in the previous section will be modified, such that range direction is set to be parallel to the x-axis to adapt to the settings in the electromagnetic field simulation software.

### 3.1. Monostatic Case

In this case, electromagnetic field simulation data of specific scenarios, calculated with the method of moment (MoM), will be utilized to test the performance of the three algorithms, including their applicability and efficiency. The parameters used in these two simulations are listed in Table 1.

**Figure 5:**
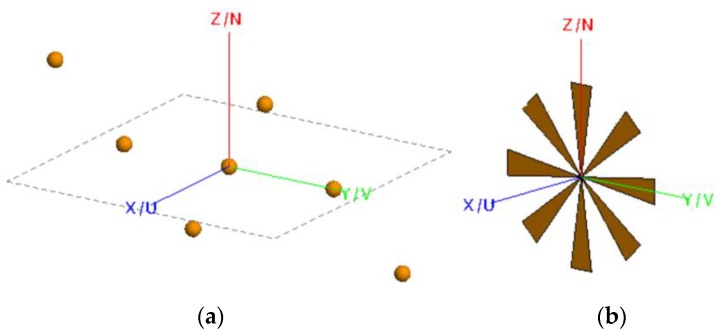
Two kinds of targets used in the simulations: (**a**) Seven extremely small metal points that are arranged in a specific way; (**b**) Metal fan with eight blades.

Two kinds of targets have been used to reconstruct images as demonstrated by Figure 5. The first one aims to test the performance of positioning and focusing, and the second tests the ability of the algorithms to present objects with complex shapes.

In Figure 5a, seven metal points that are extremely small (the radii are about a thirtieth of the center wavelength) are used as ideal scatter point targets. They are located in the coordinate (0, 0, 0), (0, 0.03, 0), (0, −0.03, 0), (0.03, 0.03, 0.03), (0.03, −0.03, 0.03), (−0.03, 0.03, −0.03), (−0.03, −0.03, −0.03). The unit used in the ordinate is meter. These points distribute mainly on three planes, located at *x* = 0, *x* = 0.03 and *x* = −0.03, respectively.

**Figure 6:**
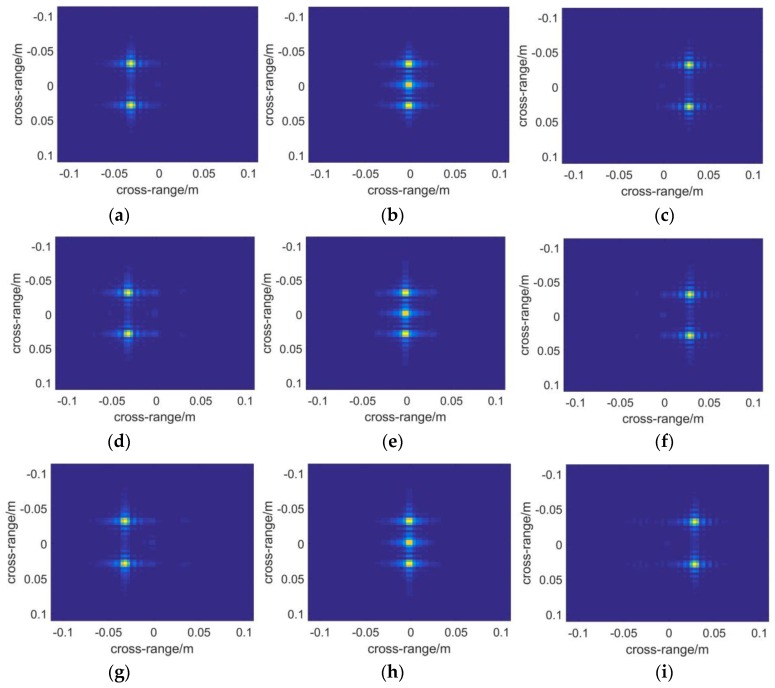
The slice figures along range direction of different distance with BPA, RMA and PMA for monostatic case respectively: (**a**) x = 0.03 with BPA; (**b**) x = 0 with BPA; (**c**) x = −0.03 with BPA; (**d**) x = 0.055 with RMA; (**e**) x = 0 with RMA; (**f**) x = −0.064 with RMA; (**g**) x = 0.03 with PMA; (**h**) x = 0 with PMA; (**i**) x = −0.03 with PMA.

In Figure 6, the results are demonstrated in the form of slice figures. The first and the third row are obtained from BPA and PMA, respectively. According to their actual coordinates, the slice positions are just where they should be. However, four points appear in error slice positions in Figure 6d–f. That means RMA’s performance of positioning in range direction is poor.

**Figure 7:**
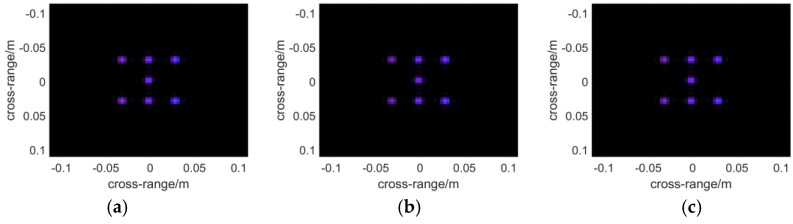
The 3-D reflectivity coefficients calculated with the algorithm of: (**a**) BPA; (**b**) RMA; (**c**) PMA. The brightness of each pixel represents the maximum modulus of the complex voxels along the range direction, while the color, which ranges from red to blue with increasing distance, of each pixel indicates the position where the maximum modulus is situated.

To demonstrate the final results in the form of a 2D image, a function is applied to present 3D complex matrices. The brightness of each pixel represents the maximum modulus of the complex voxels along the range direction, while which ranges from red to blue with increasing distance, of each pixel indicates the position where the maximum modulus is situated. Additionally, noise suppression is introduced in the function with a threshold to filter out low amplitude voxel values, which are supposed to be the noise. In the simulations, the threshold is set to be 0.1 as the noise comes mainly from sidelobes, whereas a higher threshold shall be adopted in treating experimental data. In Figure 7, the results of the algorithms are presented with the plot function. It can be seen that the left two points are nearest to the scanning plane, whose *z*-coordinate component is 0.03. While the two points on the right side whose color tends to be blue must be farther than the central ones.

**Figure 8:**
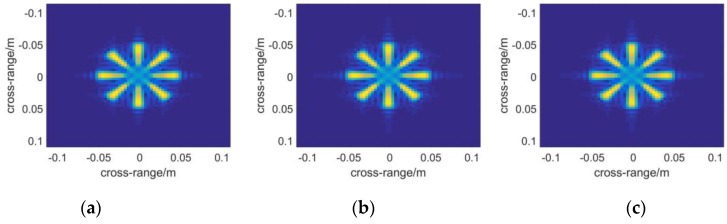
Slice figures reconstructed by the algorithm of: (**a**) BPA; (**b**) RMA; (**c**) PMA.

**Figure 9:**
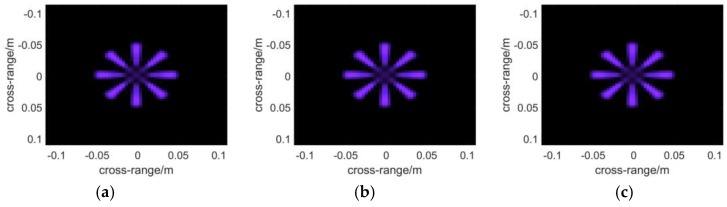
The 3-D reflectivity figures are presented which are calculated with the algorithm of: (**a**) BPA; (**b**) RMA; (**c**) PMA.

Next a metal fan is used to test the ability of each algorithm to present objects with complex shapes. The profile of the fan is shown in Figure 5b. The nearer to the central part of the model, the better resolution will be required to depict it. Therefore it reflects the imaging resolution to some extent. Figure 8 and Figure 9 demonstrate the 3D images reconstructed by the three algorithms. It seems that they have nearly the same resolution across range direction as well as the sidelobe-performance.

Finally, the efficiency of each algorithm will be discussed. Here it is presented as the processing time consumed, listed in Table 2. It can be seen that RMA achieves the highest efficiency amongst the three algorithms, while BPA takes too long to be used in a real-time system; PMA performs much faster than BPA, retaining high precision of BPA, which makes compromise between BPA and RMA.

### 3.2. Multistatic Case

In this section, the performance of PMA for the multistatic case, the procedure of which has been described in detail in Section 2.2, will be tested with electromagnetic field simulation briefly here. The metal fan in Figure 7b is used as the target, and the antenna array is arranged in the geometry shown in Figure 3a. One hundred and one transmitting and receiving elements are used in this scenario, and the operation procedure will be as follows: each transmitting element radiates signals in sequence while all the receiving elements receive signals at the same time. In fact, the plus array imaging is simpler than the case mentioned in Section 2.2, as *x_t_* = 0 and *y_r_* = 0. However, the principle of the algorithm can be proved without loss of generality.

**Figure 10:**
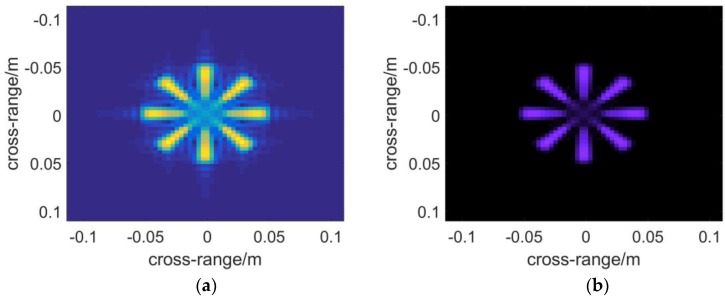
PMA applied to electromagnetic field simulation in the multistatic antenna array case: (**a**) the slice figure; (**b**) 3-D reflectivity figure.

The results of multistatic array imaging are demonstrated in Figure 10. Compared to Figure 8 and Figure 9, it is found that the performance is on par with that of the monostatic case.

## 4. Experimental Results

**Figure 11:**
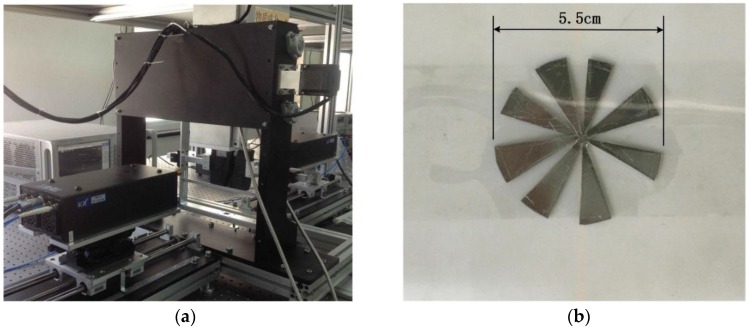
The setup of the experiment: (**a**) the experiment platform; (**b**) the sample to be imaged.

In this section, we present results of the monostatic experiment. Figure 11 demonstrates the setup of the experiment and the target to be imaged. A model N5247A network analyzer (Agilent, Santa Clara, CA, USA) is used to transmit, receive and process the signals. The signal used is a step-frequency continuous wave, and will be received after down-conversion. The signals received are analyzed in frequency domain, where it will be convenient to process. Mechanical raster scanning is realized by a 2D scanning platform. The parameters applied in this experiment are listed in Table 3.

**Figure 12:**
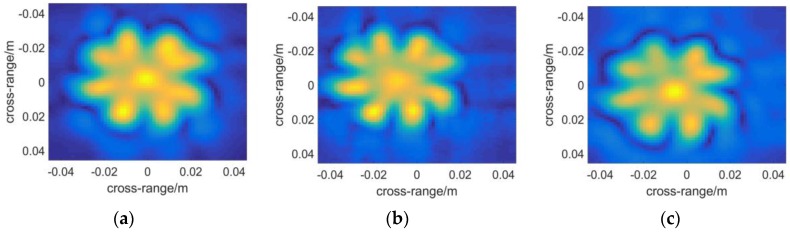
Slice figures of the metal fan at: (**a**) x = 0.398 with BPA; (**b**) x = 0.317 with RMA; (**c**) x = 0.397 with PMA.

**Figure 13:**
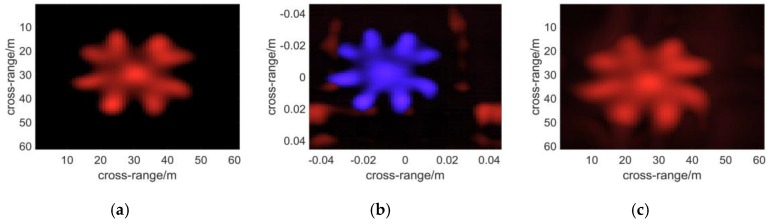
3-D reflectivity figures by: (**a**) BPA; (**b**) RMA; (**c**) PMA. The brightness of each pixel represents the maximum modulus of the complex voxels along the range direction, while the color, which ranges from red to blue with increasing distance, of each pixel indicates the position where the maximum modulus is situated.

The slice figure results in Figure 12 prove the poor positioning performance of RMA again. The slice position agrees well between Figure 12a,c, while an error of 0.08 m happens in RMA. In Figure 13, some notable cases happen in Figure 13b. Firstly, some red artifacts appear around the image. It results from the errors introduced by interpolation. In fact, the poor positioning performance must be relevant to defocus in the range direction from this point of view.

Secondly, the color of the fan in Figure 13b tends to be blue while the other two images appear red. This is also due to the appearance of artifacts. The plot function of the 3D reflectivity coefficients will be scaled to the maximum datum after noises-removing operation. The color in an image does not refer to an absolute range but a relative quantity. Therefore the three images cannot be contrasted directly in color. It simply means that the artifacts appear nearer to the scanning aperture than the target.

## 5. Discussion

In this section, some phenomena in the simulations and experiments will be discussed further. It is known that RMA takes the advantage of FFT in all three dimensions and achieves the highest efficiency. However as the samples distribute unequally in the range direction, interpolation called “Stolt interpolation [40]” should be applied to rearrange the spectrum. Thus a sinc interpolation is introduced and the error caused by the sinc interpolation is smaller than the resolution in the range direction in principle. However, as the interpolation samples are limited, slight defocus in the range direction will be introduced after all. And one of the ramifications may be its poor positioning performance. From another point of view, the reason may be explained in the following way: the sinc interpolation is conducted by moving data in wavenumber domain along the range direction with a sinc weighting function, thus the position tends to shift. Another problem is the artifacts appearing in Figure 13b. However, it is noticed that in the results of the simulations (Figure 9b) the artifacts do not appear. No noise is included in the simulations, while it will certainly appear in the experimental data. As interpolation causes defocus, the signals will be added incoherently in some voxels. Thus peaks may appear in some unexpected positions that are considered as artifacts.

The algorithm of BP is very classical. Its principle is simple and easy to understand. Recently some modified BPAs have been proposed, which aim to accelerate. However, they are not yet suitable for real-time systems. Whereas BPA processes the echoes directly without any approximation, it is supposed to be closest to ideality in resolution. Thus in this article the result of BPA is introduced as a criterion of image quality. Furthermore, it is preferable to mesh the grid more densely which will introduce more computation.

In fact, Phase-shift Migration Algorithm relates to RMA and BPA internally. It applies Equation (7) directly without carrying out interpolation. Thus it retains the performance of BPA, but operates a little slower than RMA. However, PMA can be implemented in parallel, and has the potential of real-time processing.

## 6. Conclusions

In this research, the Phase-shift Migration Algorithm (PMA) is proposed in terahertz imaging, the principle of which is derived in both monostatic and multistatic cases. The sampling criteria and spatial resolution evaluation are obtained in multistatic case. Electromagnetic field simulation and experimental results verify the applicability and performance of PMA. Compared with RMA and BPA, the features and drawbacks of PMA are summarized below:

- High precision of positioning relative to RMA, proved in the simulations and experiments;
- Capability to operate without knowing the distance a priori. In RMA, the distance from the scanning array to the objective scene center must be known before image reconstruction, or the final images may deteriorate; however this is not the case in PMA, as long as the frequency sampling interval satisfies the requirement of the scope of the objective;
- Conserve the focusing and resolution performance of BPA in principle without any approximation;
- More computation intensive than RMA, though can be implemented in parallel;
- Must operate in equal-interval sampling case. This is a fatal restriction, especially in multistatic cases. But BPA will not suffer from the same.

This algorithm has the potential of working in multiple arrays. The principle of the idea can be found in reference [14], and our future work may focus on the algorithm used in the scenario of multistatic case, especially in sparse arrays.

## Figures and Tables

**Table 1:** The parameters used in simulations.

Parameters	Value
Operation frequency range	75 GHz–110 GHz
Number of frequency samplings	101
Distance from antenna to *y*-*z* plane	1 m
Scanning aperture size in the *y*/*z* direction^1^	0.441 m
Lateral sampling interval	0.0035 m

^1^ Both directions across range have the same scanning aperture.

**Table 2:** Time consumed by each algorithm.

Algorithm	Time Consumed (s)
BPA	4430.7
RMA	2.4
PMA	7.3

**Table 3:** The parameters used in the experiment.

Parameters	Value
Operation frequency range	75 GHz~110 GHz
Number of frequency samplings	201
Antenna 3 dB bandwidth	10.8°
Distance from target to scanning array	0.36 m
Space samplings interval	0.0015 m
Number of samplings across range^1^	61

^1^ Both directions across range have the same number of samplings.

## Appendix A

In this appendix we outline the derivation of sampling criteria and spatial resolution. In the array imaging case, the path of the electromagnetic wave is depicted in Figure 3b, and expressed as:

(A1) $$\begin{array}{cc} R\left(x,y,z\right) & =\sqrt{{\left(x-x_{t}\right)}^{2}+{\left(y-y_{t}\right)}^{2}+{\left(z-z_{t}\right)}^{2}}+\sqrt{{\left(x-x_{r}\right)}^{2}+{\left(y-y_{r}\right)}^{2}+{\left(z-z_{r}\right)}^{2}}\\ & =R_{t}\left(x,y,z\right)+R_{r}\left(x,y,z\right) \end{array}$$

Taking the partial derivative of R(x,y,z) with respect to x_t_ results in:

(A2) $$\frac{\partial R\left(x,y,z\right)}{\partial x_{t}}=\frac{x_{t}-x}{\sqrt{{\left(x-x_{t}\right)}^{2}+{\left(y-y_{t}\right)}^{2}+{\left(z-z_{t}\right)}^{2}}}=\frac{x_{t}-x}{R_{t}\left(x,y,z\right)}$$

If a step-frequency continuous wave is used as the excitation source, the phase of the received signal will be:

(A3) $$\varphi \left(x_{t}\right)=-\frac{2\pi }{\lambda }R\left(x,y,z\right)$$

Supposing ∆x is the sample interval, the Nyquist sampling theorem dictates:

(A4) $$\left|\frac{\partial \varphi \left(x_{r}\right)}{\partial x_{t}}\right|\Delta x_{t}\leq \pi$$

Now suppose the planar antenna array’s aperture size in the *x*-direction is *L_x_*, and the objective domain’s scope in the *x*-direction is *D_x_*. When the transmitting and receiving elements are chosen to be on the edge of the object box in the x-direction, and the scatter point is exactly on the other side of the edge, the left side of Equation (A4) reaches its maximum. Thus the sampling criteria in the *x*-direction can be obtained as:

(A5) Δxt≤λmin(Lxt+Dx)24+R02Lxt+Dx

where *L_xt_* refers to the array’s aperture size of the transmitting elements in the *x*-direction, and *R*_0_ is the nearest distance between the antenna array and the target’s front surface. In the same manner the other sampling criteria in the *x*-direction and the *y*-direction can be concluded:

(A6) Δxr≤λmin(Lxr+Dx)24+R02Lxr+Dx

(A7) Δyt≤λmin(Lyt+Dy)24+R02Lyt+Dy

(A8) Δyr≤λmin(Lyr+Dy)24+R02Lyr+Dy

Derivation of spatial resolution: From the spectral point of view, the resolution $\rho_{u}$ in the *u*-direction (*u* can be *x*, *y* or *z*) relates to the support domain of wavenumber $\Delta k_{u}$:

(A9) $$\rho_{u}=\frac{2\pi }{\Delta k_{u}}$$

In this regard, the resolution in the range direction and the lateral directions can be obtained as:

(A10) ρr=2π4πBc=c2B

(A11) $$\rho_{a}=\frac{2\pi }{\Delta k_{a}}$$

where $\rho_{r}$ and $\rho_{a}$ (the subscript a can be x or y) mean the resolution along and across the range direction, while B is the bandwidth of the signal used. *c* represents the travelling speed of electromagnetic wave in free space. As $k_{x}$ is calculated from:

(A12) $$\begin{array}{cc} k_{x} & =\frac{d\varphi \left(x\right)}{dx}=-\frac{2\pi }{\lambda }\left[\frac{x-x_{t}}{R_{t}\left(x,y,z\right)}+\frac{x-x_{r}}{R_{r}\left(x,y,z\right)}\right]\\ & \approx -\frac{2\pi }{\lambda_{c}}\frac{2x-x_{t}-x_{r}}{R_{0}} \end{array}$$

$\Delta k_{x}$ can be obtained as:

(A13) $$\Delta k_{x}=\frac{2\pi }{\lambda_{c}R_{0}}\left(L_{xt}+L_{xr}\right)$$

Thus we will get the lateral resolution along the *x*- and *y*-direction:

(A14) $$\rho_{x}=\frac{\lambda_{c}R_{0}}{L_{xt}+L_{xr}}$$

as well as:

(A15) $$\rho_{y}=\frac{\lambda_{c}R_{0}}{L_{yt}+L_{yr}}$$
